# The Role of microRNAs in Alzheimer’s Disease and Their Therapeutic Potentials

**DOI:** 10.3390/genes9040174

**Published:** 2018-03-21

**Authors:** Munvar Miya Shaik, Ian A. Tamargo, Murtala B. Abubakar, Mohammad A. Kamal, Nigel H. Greig, Siew Hua Gan

**Affiliations:** 1 School of Medical Sciences, Universiti Sains Malaysia, Kubang Kerian 16150, Malaysia; munvar.shaik@gmail.com; 2 Drug Design and Development Section, Translational Gerontology Branch, Intramural Research Program, National Institute on Aging, National Institutes of Health, Baltimore, MD 21224, USA; iatamargo@gmail.com (I.A.T.); greign@mail.nih.gov (N.H.G.); 3 Department of Physiology, Faculty of Basic Medical Sciences, College of Health Sciences, Usmanu Danfodiyo University, PMB 2254 Sokoto, Nigeria; murtala.bello@udusok.edu.ng; 4 Metabolomics and Enzymology Unit, Fundamental and Applied Biology Group, King Fahd Medical Research Center, King Abdulaziz University, P.O. Box 80216, Jeddah 21589, Saudi Arabia; prof.ma.kamal@gmail.com; 5 School of Pharmacy, Monash University Malaysia, Jalan Lagoon Selatan, Bandar Sunway 47500, Selangor, Malaysia

**Keywords:** miRNAs, Alzheimer’s disease, BACE1 inhibitors, γ-secretase inhibitors

## Abstract

MicroRNAs (miRNAs) are short, endogenous, non-coding RNAs that post-transcriptionally regulate gene expression by base pairing with mRNA targets. Altered miRNA expression profiles have been observed in several diseases, including neurodegeneration. Multiple studies have reported altered expressions of miRNAs in the brains of individuals with Alzheimer’s disease (AD) as compared to those of healthy elderly adults. Some of the miRNAs found to be dysregulated in AD have been reported to correlate with neuropathological changes, including plaque and tangle accumulation, as well as altered expressions of species that are known to be involved in AD pathology. To examine the potentially pathogenic functions of several dysregulated miRNAs in AD, we review the current literature with a focus on the activities of ten miRNAs in biological pathways involved in AD pathogenesis. Comprehensive understandings of the expression profiles and activities of these miRNAs will illuminate their roles as potential therapeutic targets in AD brain and may lead to the discovery of breakthrough treatment strategies for AD.

## 1. Introduction

As of 2015, 47 million people worldwide are estimated to be living with dementia, 9.9 million people are estimated to be diagnosed with dementia each year, and the global costs of the disease are estimated to be 818 billion United States dollars (USD$). Due to increasing life expectancies worldwide, the prevalence of dementia is projected to double every 20 years, meaning that 131 million people will live with dementia by the year 2050. The global costs of the disease will exceed USD$ 2 trillion by 2030.

The symptoms of dementia can result from a number of neurological disorders and neurodegenerative diseases. Alzheimer’s disease (AD) is the most common cause. Recent data suggest that AD is the sole cause of dementia in 38% of all elderly patients, while combinations of AD and other forms of dementia have been estimated to be the cause in an additional 48% of elderly patients, meaning that AD pathology may be involved in approximately 86% of all the cases of dementia in the elderly [1]. A previous estimate suggested that AD is the sole cause of dementia in 60% of the demented elderly population and is involved in mixed pathology in an additional 17%, meaning AD is involved in 77% of elderly dementias [2].

Dementia caused by AD is broadly characterized by the deterioration of memory and cognition with age. As the National Institute on Aging and Alzheimer Association’s 2011 revised guidelines for the diagnosis of AD suggest, AD may be separated into three stages of disease progression: preclinical AD, mild cognitive impairment (MCI) due to AD, and dementia due to AD [3,4,5,6]. Individuals with preclinical AD manifest alterations in the levels of biomarkers that are related to AD in their blood, cerebrospinal fluid (CSF), and brain, but do not display impaired memory or cognition. Individuals with MCI due to AD have some memory loss and problems with cognition that are apparent to their closest acquaintances, but do not interfere with their day-to-day life. Finally, individuals with dementia due to AD are incapacitated due to severely impaired cognition and extensive memory loss.

AD is a sporadic, multifactorial disease with a number of well-studied risk factors. Age is the most significant risk factor for the development of AD. The population of AD patients over the age of 65 has been estimated to be 96% [7,8]. After age 65, the incidence of AD doubles every five years. In fact, 11% of all people age 65 and older have AD, while 32% of all people age 85 and older have the disease [7]. Family history of AD is an important risk factor as well, though autosomal-dominant genetic mutations that guarantee the development of the disease are found in less than 1% of patients diagnosed with AD [9]. Inheritance of the ε4 polymorph of apolipoprotein E (ApoE-ε4) also greatly increases one’s risk of developing AD [10,11]. Additionally, cardiovascular stress resulting from obesity, diabetes, hypercholesterolemia, and hypertension, among other things, increases an individual’s likelihood of developing AD [12,13,14,15,16].

Though the precise cause or causes of AD have not been identified, many of the salient characteristics of the disease at the cellular and molecular levels have been described in detail. At the cellular level, AD is characterized by the progressive loss of neurons in the cerebral cortex, as well as several other subcortical brain areas. Reduced numbers of synapses and synaptic dysfunction are observed in affected regions, as well. The deterioration and death of neurons in affected regions leads to atrophy of the AD brain [17].

At the molecular level, AD is characterized by overproduction of the 40 and 42 amino acid isoforms of β-amyloid (Aβ_1–40_ and Aβ_1–42_), which form insoluble, extracellular aggregates of Aβ called *amyloid plaques*, and by the hyperphosphorylation and aggregation of the microtubule-associated protein tau within neurons. These insoluble, intracellular aggregates are called neurofibrillary tangles (NFTs). Neuroinflammation is also responsible for neuron and synapse loss in AD. Although amyloid plaques, NFTs, and neuroinflammation have been hypothesized to be neurotoxic and responsible for the loss of neurons and synapses in AD brain, the precise cause and sequence of AD pathogenesis remains controversial. The focus of the current article is to evaluate the presently known role of microRNAs (miRNAs) in the development and progression of AD, as miRNAs are known to regulate an extensive network of cellular programmes—dysfunction of which has been linked to a broad number of diseases, including neurodegenerative ones. To understand the relevance of select miRNAs in the processes that underpin AD, we first overview the mechanisms that underlie the primary pathologies of AD (amyloidogenesis, tauopathy, neuroinflammation and synaptic dysfunction), then provide a synopsis of key drug therapies developed and in development for AD. In the context of this information, we then review ten widely studied dysregulated miRNAs in AD, concentrating on their altered expressions in AD (vs. normal healthy aged brain) and their participation in pathways that appear to contribute to AD pathology. Lastly, we overview several drug classes with potential to target and manipulate miRNA activity, and thus hold promise as future therapeutics for AD treatment.

### 1.1. Amyloidogenesis

Aβ_1–42_ is generated by the sequential proteolytic cleavage of amyloid precursor protein (APP), a type I transmembrane glycoprotein whose primary function is unknown. To generate Aβ_1–42_, APP is first cleaved in its extracellular domain by β-site APP cleaving enzyme 1 (BACE1), also known as β-secretase, which has been implicated in myelin sheath formation in peripheral neurons [18]. This cleavage releases soluble APP β (sAPPβ) into the extracellular space and leaves the remaining 99 amino acid carboxy-terminal fragment (CTF99) of APP that is embedded in the cell membrane. CTF99 is then cleaved by the γ-secretase complex within the lipid bilayer of the cell membrane at the 42nd amino acid relative to the BACE1 cleavage site. The γ-secretase complex is a multi-subunit, intramembrane protease putatively comprising anterior pharynx-defective 1a or b (APH-1a or APH-1b), nicastrin (NCSTN), presenelin 1 or 2 (PS1 or PS2), and presenelin enhancer 2 (PEN-2) [19]. This cleavage releases Aβ_1–42_ into the extracellular space and the 49–50 amino acid APP intracellular domain (AICD) into the cytoplasm.

The 40 amino acid isoform of Aβ (Aβ_1–40_), which is less closely correlated with cognitive decline in AD than Aβ_1–42_, is generated in a similar manner. The difference is that CTF99 is cleaved by γ-secretase at the 40th amino acid from the BACE1 cleavage site to generate Aβ_1–40_. Additionally, intracellular generation of Aβ_1–40_ has been shown to occur in the trans-Golgi network, whereas intracellular generation of Aβ_1–42_ has been shown to occur in the endoplasmic reticulum-Golgi intermediate compartment [20,21,22]. Though Aβ_1–40_ and Aβ_1–42_ are the most prevalent isoforms of Aβ in AD, several other isoforms of Aβ have been observed that range from 36 to 43 amino acids.

It is important to note that APP cleavage by one of several α-secretases prohibits the generation of Aβ. α-secretases are members of the A disintegrin and metalloprotease (ADAM) family of sheddase proteins that cleave APP at its α site, which is located within the Aβ sequence. ADAM9, 10, 17, and 19 have all demonstrated α-secretase activity [23,24]. α-secretase cleavage of APP releases soluble APP α (sAPPα) into the extracellular space, and leaves the remaining 83 amino acid fragment of APP (CTF83) embedded in the cell membrane. Subsequent cleavage of C83 by γ-secretase discharges the non-pathogenic p3 peptide into the extracellular space, leaving behind the AICD.

Another significant difference between amyloidogenic and non-amyloidogenic processing of APP is that only amyloidogenic processing occurs on lipid rafts. It has been shown that α-secretases are more likely to interact with APP in non-rafted areas of the plasma membrane, whereas BACE1 is more likely to interact with APP within lipid rafts in the plasma membrane [25]. The increased likelihood of amyloidogenic APP processing in the presence of high densities of lipid rafts in the cell membrane has been hypothesized to explain the increased risk of AD in individuals who encode the ApoE-ε4. This hypothesis suggests that ApoE-ε4 may increase the number of lipid rafts in cell membranes due to the lower turnover of cholesterol as compared to other polymorphs of ApoE [26]. This hypothesis also helps to explain the increased risk of AD in individuals with hypercholesterolemia.

Aβ_1–40_ and Aβ_1–42_ are believed to lack secondary and tertiary structures. Importantly, the two additional amino acids on the carboxy terminus of Aβ_1–42_ have been shown to be involved in nucleation of amyloid aggregates [27]. Increased levels of Aβ_1–42_ have thus been shown to increase the rate of plaque formation in solution. Accordingly, the genetic mutations in APP, PS1, and PS2 observed in familial AD (FAD) have been shown to increase the ratio of Aβ_1–42_ to Aβ_1–40_ [28,29,30]. The Aβ_1–42_:Aβ_1–40_ ratio, as opposed to the total amount of Aβ, has been shown to be a stronger indicator of AD, with greater ratios correlating with increased probability of AD as opposed to other forms of dementia [31]. It is important to note that although the insoluble amyloid plaques observed in AD brain were long speculated to be the cause of neurotoxicity, it is now believed that the levels of soluble Aβ_1–42_ oligomers (2-6 peptides) are more likely the cause of neuronal loss in AD [32].

### 1.2. Tauopathy

Tau is a microtubule-associated protein involved in the assembly and stabilization of microtubules. It has six isoforms resulting from alternative splicing in humans [33]. Tau is primarily expressed in neurons of the central nervous system (CNS). Within neurons, tau is predominantly found in the distal regions of axonal microtubules. It is not commonly found in dendritic microtubules [34]. Tau is hyperphosphorylated in AD brain. Glycogen synthase kinase 3 β (GSK3β), cyclin-dependent kinase 5 (Cdk5), cAMP-dependent protein kinase (PKA), and microtubule-affinity-regulating kinase (MARK) are most likely responsible for the pathological phosphorylation of tau [35]. Various phosphatases are likely involved, as well. In AD, tau dissociates from microtubules in response to hyperphosphorylation and the insoluble hyperphosphyrlated tau monomers form paired helical filament (PHF) dimers. Paired helical filaments ultimately aggregate to form the intracellular NFTs that have been a hallmark of AD histopathology since Alois Alzheimer first observed them in 1906.

Tauopathy in AD is commonly believed to be a result of amyloidogenesis, rather than a distinct event in the pathogenesis of the disease [36,37,38]. Additionally, it is unclear whether hyperphosphorylated tau and its aggregated forms are neurotoxic in and of themselves, or whether the lack of microtubule-bound tau causes synaptic and neuronal dysfunction and death via destabilization of the cytoskeleton.

### 1.3. Neuroinflammation

Chronic inflammation is observed in the brains of AD patients. Pathological aggregates of misfolded protein, such as amyloid plaques, Aβ oligomers and NFTs, as well as neuronal and synaptic signals of degradation are believed to be the main triggers of the inflammatory response in AD. Inflammation in AD brain is primarily mediated by microglia and astrocytes. Activated microglia and reactive astrocytes release proinflammatory cytokines, as well as proteolytic enzymes upon exposure to AD-associated antigens. Tumor necrosis factor (TNF)-α, interleukin (IL)-1β and -6, as well as transforming growth factor (TGF)-β are among the most highly upregulated inflammatory cytokines detected in AD brain [39]. Of relevance to AD, activated microglia and reactive astrocytes have been shown to upregulate proteolytic enzymes that exacerbate AD pathology, such as BACE-1 and γ-secretase [40,41]. Additionally, increased expression of APP has been associated with neuroinflammation. Multiple studies have shown increased the production of APP, γ-secretase, BACE-1 and consequently Aβ in neurons exposed to neurotoxic concentrations of Aβ, which suggests that the neuroinflammatory response in AD facilitates a positive feedback mechanism for the production of Aβ [39]. Therapeutic intervention into the process of chronic neuroinflammation in AD may play a key role in slowing the progression of the disease.

### 1.4. Synaptic Dysfunction

Synapse loss is more highly correlated with severity of dementia than either amyloid plaque or NFT density is, and is in fact the best correlate with cognitive impairment in dementia [42,43,44,45,46]. Synapse loss begins early in the progression of AD and is invariably observed in AD brain. An extensive body of literature suggests that synapse loss is the key cellular event that is responsible for memory impairment in AD. For example, Geinisman et al. released a series of publications in the late 1980s and early 1990s showing that aged rats that performed poorly on memory-based behavioral paradigms had significantly reduced numbers of perforated synapses in the hippocampal formation and dentate gyrus, and significantly reduced numbers of axospinous synapses in the dentate gyrus as compared to both young and aged rats that performed well on the memory-based tasks [47,48,49]. The greater significance of the quantity of hippocampal synapses rather than neurons is reinforced by several studies showing that the numbers of hippocampal neurons are not significantly altered between rats with different performances on memory-based tasks [50,51]. In humans, a number of studies have shown reduced quantities of synapses and synaptic protein expression in several areas of AD brain, and have demonstrated the strong correlation between cognitive impairment and synapse loss [43,46,52,53].

Soluble Aβ_1–42_ oligomers are believed to be the primary cause of synapse dysfunction and loss in AD [54]. A number of studies have demonstrated the harmful effects of these oligomers on synaptic processes. For example, one cell culture study in APP-overexpressing Chinese hamster ovary (CHO) cells showed that Aβ oligomers cause significant reductions in long-term potentiation (LTP), an indicator of synapse strength and activity, which is believed to be a critical component of memory and learning at the cellular level [55]. These reductions in LTP were abolished by the inhibitors of Aβ oligomerization. An earlier study in mice demonstrated an inverse correlation between levels of Aβ_1–42_ and density of synaptophysin-immunoreactive (SYN-IR) presynaptic terminals in human APP (hAPP) transgenic mouse brains [56]. These studies contribute to an extensive body of evidence suggesting that oligomeric Aβ_1–42_ is responsible for the synaptic dysfunction and loss observed in AD [57,58].

The damages that Aβ inflicts on synapses in AD brain are produced by a number of pathological effects. For example, Aβ has been shown to induce endocytosis of *N*-methyl-d-aspartate receptors (NMDArs), which are key receptors involved in synaptic plasticity and memory function [59], in the postsynaptic membranes of cortical neurons [60]. Similarly, Aβ caused endocytosis of synaptic α-amino-3-hydroxy-5-methyl-4-isoxazolepropionic acid receptors (AMPArs) in cell culture [61], another receptor integral to synaptic plasticity and transmission. Aβ_1–42_ also binds the α-7 nicotinic acetylcholine receptor (nAChR) thus impairing cholinergic transmission and LTP [62]. Oligomeric Aβ_1–42_ also impedes synaptic function and survival by reducing the transcription of brain-derived neurotrophic factor (*BDNF*) [63].

## 2. Current Drug Therapies for Alzheimer’s Disease

### 2.1. BACE1 Inhibitors

Research into BACE1 inhibitors began in the 1990s. BACE1 was selected as a target for inhibition because it initiates amyloidogenesis, and is thus likely the rate-limiting enzyme in Aβ_1–42_ synthesis. Beginning in 1999 with the introduction of P10-P4′ StatVal, a series of BACE1 inhibitors were produced in academia and industry that improved upon this pioneering compound by generating lower IC_50_ values, improving permeability across the blood-brain barrier (BBB), and enhancing oral bioavailability [64,65]. Of note, CoMentis compound CTS-21166 was well-tolerated by Phase I trial volunteers and reduced Aβ production by up to 80%, Merck compound MK-8931 was similarly well-tolerated and reduced CSF Aβ_1–40_, Aβ_1–42_, and sAPPβ by up to 90%, Eli Lilly compound LY2886721 showed promising results in Phase I trials, and Biogen’s BACE1 inhibitior, E2609 reduced plasma Aβ levels in healthy adults in Phase I trials [66,67,68,69]. Currently, the status of CTS-21166 is confidential—but, notably, the agent does not appear listed on the company’s Research & Development pipeline website. Phase III clinical studies of MK-8931 in prodromal (early) AD were halted by Merck in February 2018 on the recommendation of the external Data Monitoring Committee concluding that it was unlikely that positive benefit/risk could be established if the trial continued. The development of LY2886721 has been terminated due liver abnormalities in several patients, and E2609 is in Phase III trials in a joint venture between Biogen and Eisai, with data expected in June and September 2020.

### 2.2. γ-Secretase Inhibitors and Modulators

γ-secretase inhibitors (GSIs), which target the second proteolytic enzyme that is responsible for the generation of Aβ_1–40_ and Aβ_1–42_, were also first developed in the 1990s. These compounds, which mostly target the PS1 and 2 subunits of γ-secretase, effectively reduced Aβ production in cell and rodent models of disease [70]. However, upon the clinical investigation of multiple GSIs developed by companies, such as Merck, Eli Lilly, and Bristol-Meyers Squibb, among others, these compounds were revealed to be both ineffective and unsafe due to side effects resulting from off-target inhibition of Notch 1 signaling. As a result, investigation into GSIs as AD therapeutics has largely come to halt. Currently, γ-secretase modulators (GSMs), which specifically block protease activity at the γ-secretase cleavage site on APP, are the current focus of γ-secretase-targeted therapies [71,72]. Their development, however, has been hampered by ineffective clinical trials, which produced similar results to those of the failed BACE1 inhibitor trials.

### 2.3. Aβ Immunotherapy

Immunotherapeutic approaches to reducing Aβ expression have also been investigated. Similar to the BACE1 inhibitors and GSIs, research into both active and passive vaccinations against Aβ began in the late 1990s [73]. Elan and Wyeth’s vaccine, AN1792 was the first active vaccination to undergo clinical trials in AD. The trials were stopped during Phase II due to encephalitis in a number of patients. The available data from the trials show that the vaccine effectively reduced Aβ levels, but did not affect cognitive changes [74]. Since this pioneering trial, clinical trials for multiple vaccines from companies, such as Novartis and Pfizer, have been initiated, most of which are currently in Phase I or II [75,76].

Clinical trials on passive vaccinations against Aβ constitute a broader and more developed area of immunotherpeautic research. Multiple passive vaccinations have progressed to Phase III clinical trials, however none have proved effective in treating the cognitive deficits experienced in AD [73]. Recently Eli Lilly reported on its vaccine Solaneuzumab, which showed no significant effect on cognition in the overall population it examined for its Phase III trial, but did show some benefits for the younger participants in the study upon post hoc analysis of the data [77]. The results of the Solaneuzumab trial underscore one of the primary explanations for the failures of both Aβ vaccinations and inhibitors of amyloidogenesis: namely, that these interventions are only effective when given to presymptomatic patients. This hypothesis will be tested as Solaneuzumab undergoes an additional Phase III trial in pre-MCI subjects.

### 2.4. Acetylcholinesterase Inhibitors

Four acetylcholinesterase (AChE) inhibitors have been approved for the treatment of AD. The four AChE inhibitors, Tacrine, Donepezil, Rivastigmine and Galantamine were approved by the Food and Drug Administration (FDA) in 1993, 1996, 2000, and 2001, respectively. Tacrine was discontinued in the US in 2013 due to safety issues. These compounds inhibit the breakdown of synaptic acetylcholine (ACh) by AChE, thus enhancing the beneficial effects of this neurotransmitter on sensory perception, attention, and plasticity. The AChE inhibitors are therefore palliative treatments for the cognitive deficits that manifest in AD, and, although sometimes indicated to delay the progression of the disease [78]—this remains controversial.

### 2.5. NMDA Receptor Antagonists

The *N*-methyl-d-aspartate (NMDA) receptor antagonist Memantine is the only drug approved for the treatment of AD in the US besides the four AChE inhibitors previously mentioned [79]. The FDA approved Memantine for the treatment of AD in 2003. The drug works by reducing the effects of glutamate excitoxicity in diseased brains via competitive inhibition of the NMDA receptor, which mediates glutamate excitotoxicity. Though Memantine reduces neuronal apoptosis in key areas of AD brain, it has a minimal effect on the progression of AD, which is similar to the AChE inhibitors.

## 3. miRNAs in Alzheimer’s Disease

MicroRNAs (miRNAs) are small non-coding RNA molecules that regulate post-transcriptional gene expression. The process of miRNAs synthesis begins in the nucleus under the influence of RNA polymerase II which give rise to primary miRNA precursor, which is subsequently processed to yield another miRNA precursor (pre-miRNA) of about 60 to 100 nucleotides. The pre-miRNA is then transferred to the cytoplasm where it further converted to a final mature miRNA. Currently, there are approximately 2650 mature human miRNAs [80].

### 3.1. Rationale for miRNA Investigation in AD

The three AChE inhibitors and one NMDA receptor antagonist that are currently used for the treatment of AD in the US are palliative medications that temporarily alleviate the cognitive symptoms of AD. There is thus an unmet need for treatments that improve upon the current medications by addressing the underlying causes of the disease. The drugs that have targeted the processes underlying AD, as described by the amyloid cascade hypothesis, however, have not demonstrated efficacy in numerous clinical trials. These disappointing results may very well be due to the relatively late stage at which these treatments are given to patients with AD, and may be improved by clinical trials with patients in earlier stages of AD progression. Having investigated BACE1 inhibitors, GSIs, and Aβ immunotherapy for approximately 20 years, however, it is prudent at this point to investigate alternative pathologies that may introduce compelling new drug targets.

One such pathology is studied in the nascent field of miRNA dysregulation in AD. This field, which came into existence after several pioneering studies were published in 2007 and 2008, is directed toward identifying miRNAs that are differentially expressed in AD brain, as compared to normal elderly controls, and determining the activities of these dysregulated miRNAs in the hopes of identifying miRNAs whose dysregulation initiates or exacerbates pathogenic mechanisms involved in AD. Here, we review ten commonly researched dysregulated miRNAs in AD, with a particular focus on their relative expressions in AD as compared to normal brain, and their involvement in pathways and mechanisms that contribute to AD pathology. Finally, we briefly discuss several classes of drugs that have been designed to target and manipulate miRNA activity and are thus potential therapeutics for the treatment of AD.

### 3.2. miRNA Biogenesis and Function

miRNAs are ~22 nt, non-coding RNAs that have emerged as critical regulators of gene expression, and have been shown to affect a multitude of cellular processes, including proliferation, differentiation, survival, and motility [81]. miRNA production and activity are complex processes. After transcription of the miRNA genes by RNA polymerase II or III, as well as cleavage of the transcripts by Drosha/DGCR8 in the nucleus, 70–100 nt pre-miRNAs are exported into the cytoplasm by Exportin 5 and cleaved by Dicer to produce ~22 nt mature miRNAs. Mature miRNAs often have multiple isoforms, which generally differ by one nucleotide and are denoted by the addition of the letter a, b, c, and so on, to the name of the miRNA. They are a miRNA family because they share the seed region by which the miRNAs bind to RNA targets. Upon entering the large, cytoplasmic multiprotein complex named RNA-induced silencing complex (RISC), mature miRNAs regulate gene expression by binding to complementary sequences on their target mRNAs, which are generally located within the 3′ untranslated regions (3′-UTRs) of the target transcripts [82]. As a result, the targeted mRNAs are either degraded or are made incapable of being translated into proteins in the cells ribosomes.

On average, a given miRNA regulates several hundred transcripts, which encode proteins that act within multiple cellular pathways and networks. Additionally, mRNA transcripts may be regulated by multiple miRNAs [83,84,85]. Due to their wide array of mRNA targets, miRNAs are able to regulate various cellular programmes, and are therefore important. Their importance in the epigenome is further demonstrated by the fact that the miRNA biogenesis pathway is highly conserved, as are many miRNA sequences and their target binding sites [86,87].

### 3.3. miRNA Dysregulation in Alzheimer’s Disease

To date, significant differences in the miRNA expression profiles of numerous diseased tissues have been detected when compared with normal tissue. These alterations have been observed in cancer, obesity, diabetes, and inflammation, neurological disorders, such as Parkinson’s disease, cardiovascular, and autoimmune diseases. Many of the pathways and mechanisms found to be influenced by dysregulated microRNAs in cancer were known to be pathological in AD as well [88]. As such, research into microRNA dysregulation in AD began after years of cancer microRNA research in the 1990s and early 2000s. As described by several pioneering studies published in 2007 and 2008, the expressions of hundreds of miRNAs are altered in AD brain. A significant portion of AD literature has since been devoted to identifying and verifying AD-relevant targets of dysregulated miRNAs [89,90]. Here, we review ten of the most extensively researched dysregulated miRNAs in AD with a particular focus on their relative expressions in AD as compared to normal brain, and their involvement in pathological mechanisms.

#### 3.3.1. microRNA-9

Several publications in the 2000s identified a brain-specific microRNA (miR-9) besides its role in connective, embryonic and nervous tissue in human or in hematopoietic nervous, endocrine, reproductive, and respiratory tissues (in mouse). In a 2002 publication by Lagos-Quintana et al., miR-9 expression was detected in tissue samples from the cerebellum, cortex, and midbrain of 18.5-week-old BL6 mice, but not in tissue samples from the heart, liver, spleen, small intestine, or colon [91]. Two years later, Sempere et al. detected miR-9 and -9* expression in the brain of six-week-old C57BL/6 mice, but not in the liver, heart, skeletal muscle, lung, kidney, or spleen [92]. Human miR-9 and -9* expression was also assayed and found in tissue from the brain, but not in tissue from the liver, heart, or skeletal muscle. Brain-specific expression of miR-9 and -9* was again observed in balb/c mice by Bak et al. in 2008, though both miRNAs were found to be expressed at low levels in the pituitary as compared to the rest of the mouse brain [93].

As the body of research indicating brain-specific expression of miR-9 continued to grow, a number of AD-related microRNA studies investigated miR-9. miR-9 was first observed to be up-regulated in AD hippocampal cornu ammonis (CA) 1 tissue as compared to late adult control tissue via DNA array and Northern gel analyses by Lukiw in 2007 [94]. In a separate study, Lukiw and Pogue observed elevated levels of miR-9 in primary culture human neural cells treated with a reactive oxygen species (ROS)-inducing dose of aluminium and iron, an in vitro model designed to simulate the environment of neural cells in AD [95].

These results were disputed by two subsequent studies published in 2008. Cogswell et al. observed reduced expressions of miR-9 in Braak III, IV, V, and VI cerebellum, hippocampus, and medial frontal gyrus as compared to those in Braak 0 to I controls [96]. Similarly, Hebert et al. reported down-regulation of miR-9 in sporadic AD cortex as compared to age-matched control cortex [97]. It is important to note that beta-site APP cleaving enzyme 1 (BACE1) and presenilin-1 (*PSEN1*) were both identified as potential targets of miR-9 in the Hebert et al. study. Only BACE1 regulation by miR-9, however, was confirmed via luciferase assay in HeLa cells, though miR-9 levels were not inversely correlated with BACE1 expression in vivo, despite a tendency toward lowered levels of miR-9 in high-BACE1 AD patients.

The down-regulation of miR-9 in AD cortex reported by Hebert et al. was consequently brought into question by a 2009 study that was published by Sethi and Lukiw [98]. In this study, levels of miR-9 in the temporal lobe neocortex of AD patients were found to be increased in comparison to those in age-matched control neocortex. Additionally, Sethi and Lukiw showed that the half-life of miR-9 is 0.8 h in brain tissue and 0.7 h in brain cells, which suggests that the discrepancies in the reports of relative miR-9 expression levels in AD brain may be due to inconsistent post-mortem intervals between patient death and brain freezing. Interestingly, miR-9 dysregulation in the temporal lobe neocortex was observed to be unique to AD among several neurodegenerative disorders, including ALS, Parkinson’s, and Schizophrenia.

Further conflicting data on miR-9 dysregulation in AD was produced by Wang et al. in 2009. The expression of miR-9 and -9* were both reduced in three-month-old APPswe/PSΔE9 mouse cerebral cortex as compared to three-month-old control mice. The expressions of both microRNAs, however, were observed to be increased in six-month-old APPswe/PSΔE9 mouse cerebral cortex as compared to six-month-old control mice. These results suggest that miR-9 may display varying expression profiles at different points in the progression of AD. The time-dependant expression profile of miR-9 may help to explain the diverse data set on miR-9 dysregulation in AD.

The debate continued in 2010 when Schonrock et al. reported decreased levels of miR-9 in APP23 mouse hippocampus as compared to non-transgenic mouse brain [99]. Decreased levels of miR-9 in primary hippocampal neurons from C57BL/6 mice treated with Aβ as compared to untreated neurons were also observed. These results provided additional evidence of reduced miR-9 expression in AD, and suggested that miR-9 regulation by Aβ may be one possible mechanism through which miR-9 down-regulation occurs.

Down-regulation of miR-9 in human AD temporal cortex as compared to healthy control, as well as enrichment in cortex grey matter relative to white matter, was observed in 2011 [100]. The following year, however, Lukiw and Alexandrov reported elevated levels of miR-9 in human AD superior temporal neocortex as compared to age-matched control [101].

Two studies were published in 2012 and 2014, respectively, which identified AD-relevant gene targets of miR-9. First, three targets of miR-9 were identified using prediction algorithms and verified via luciferase assays in HeLa cells: transforming growth factor, beta-induced (TGFBI), a protein involved in the TGF-β pathway, which controls cellular proliferation and differentiation, among other things, tripartite motif-containing 2 (TRIM2), which is a protein involved in cell protection and pathogen recognition, and whose down-regulation has been associated with early-onset axonal neuropathy [102], and sirtuin (silent mating type information regulation 2 homolog) 1 (SIRT1), a protein whose activation has been shown to have anti-aging effects and reduce Aβ production [103]. Enhanced expression of these targets would have neuroprotective effects, and as such, the authors suggest that these proteins may be upregulated in AD via reduced levels of miR-9 as a compensatory mechanism to combat cell damage [104].

Second, via luciferase assay in HEK293 cells, miR-9 was found to target calcium/calmodulin-dependent protein kinase kinase 2 (CAMKK2) [105], a protein involved in the CAMKK2-AMP-activated protein kinase (AMPK) pathway, whose activity has been shown to increase levels of phosphorylated tau (p-tau) and amyloidogenesis [106,107]. Importantly, overexpression of miR-9 in mouse primary hippocampal neurons exposed to Aβ42 insult was demonstrated to reduce levels of phosphorylated AMPK (p-AMPK) and p-tau, and increase the dendritic spine density as compared to Aβ42-exposed neurons that lacked miR-9 overexpression. Reduced levels of miR-9 would thus lead to increased neurotoxicity via the CAMKK2-AMPK pathway. These results are consistent with the reports of miR-9 down-regulation in AD.

The most recent studies on miR-9 have primarily focused on it expression profile in AD brain, as well as its potential use as a biomarker. miR-9 was observed to be down-regulated in the forebrain cortex and hippocampus of 18-month-old male Wistar rats [108]. In a separate study, New Zealand white rabbits with AD-like pathology were shown to have reduced levels of miR-9 in frontal cortex as compared to control at four weeks of AD progression [109]. The same rabbits, however, had elevated levels of miR-9 at 12 weeks of AD progression. Results in the recent studies on the use of miR-9 as a biomarker for AD have been similarly inconsistent, with one article reporting significantly reduced levels of miR-9 in human probable AD serum [110], and another reporting significantly increased levels of miR-0 in AD serum [111]. Yet another study found no significant difference between the levels of miR-9 in the plasma or CSF of AD patients as compared to control [112].

**Figure 1:**
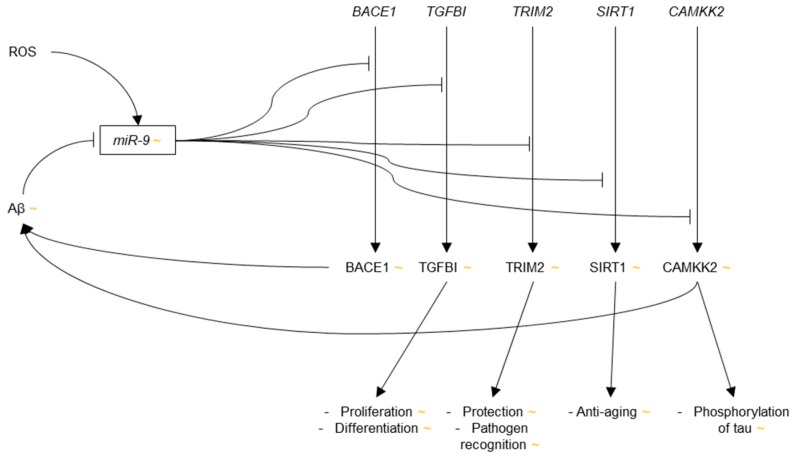
Regulation by miR-9. miR-9 inhibits the translation of β-site amyloid 1102 precursor protein cleaving enzyme 1 (BACE1), transforming growth factor, β-induced (TGFBI), tripartite motif-containing 2 (TRIM2), silent mating type information regulation 2 homolog 1 (SIRT1), and calcium/calmodulin-dependent protein kinase kinase 2 (CAMKK2). The debatable level of miR-9 expression in Alzheimer’s disease (AD) brain is indicated by the yellow tilde symbol (~), as are the hypothetical expression levels of the proteins regulated by miR-9 and their cellular effects. ROS: reactive oxygen species.

Whether the expression of miR-9 is increased or decreased in AD is debatable, but the general consensus seems to be that miR-9 is most likely down-regulated in AD. The evidence of miR-9 up-regulation in human AD brain primarily comes from Lukiw et al., whereas evidence of miR-9 down-regulation has been provided from various groups in human AD samples, as well as cell culture and mouse models of AD. Down-regulation of miR-9 would additionally be consistent with the targeting of *BACE1* and *CAMKK2* because these transcripts encode proteins that contribute to AD pathology when overexpressed. Up-regulation of miR-9 in AD, however, would be consistent with the targeting of *TGFBI*, *TRIM2* and *SIRT1* because these mRNAs produce neurotrophic proteins (Figure 1). Two studies have shown varying relative expressions of miR-9 at different points in the progression of AD, which may be an explanation for these discrepancies.

#### 3.3.2. microRNA-124

Interest in miR-124 began in 2002, when Lagos-Quintana et al. reported that miR-124 accounted for 25–48% of all miRNAs in mouse brains, and was conserved in the human genome [91]. Since this publication, much of the research into miR-124 has focused on its role in neurogenesis and neuronal development. In 2006, expression of miR-124 was shown to increase in progressive stages of neurogenesis in mouse embryonic stem cells [113]. In 2007, miR-124 was shown to be induced upon neuronal differentiation and to be constitutively expressed in differentiated neurons throughout zebrafish brain, suggesting that miR-124 may target non-neural transcripts [114]. This hypothesis was given credence by a study published around the same time by Makayev et al., which showed that miR-124 promoted brain-specific alternative splicing of mRNA transcripts [115]. miR-124 was able to influence mRNA splicing through the suppression of polypyrimidine tract binding protein 1 (PTBP1), a repressor of alternative, nervous-system-specific splicing in non-neuronal cells. Increased levels of miR-124 in differentiated neurons thus suppress PTBP1, and allow for nervous-system-specific splicing of transcripts.

miR-124 was first investigated in relation to Alzheimer’s in 2007 [94]. Lukiw found that miR-124a expression was slightly down-regulated in Alzheimer’s hippocampus as compared to age-matched controls, but the difference was not significant at the *p* < 0.05 level.

Presumably due to the lack of evidence of miR-124 dysregulation in AD, no articles were published on the subject until 2011, when Smith et al. showed that miR-124 influenced neuron-specific APP mRNA splicing, and that down-regulation of miR-124 leads to APP isoforms associated with AD [116]. To demonstrate the influence of miRNAs on neuronal APP splicing, the authors showed that the expression of non-endogenous isoforms of APP were increased in the cortex of Dicer conditional knockout mice as compared to wild type (WT) cortex. Importantly, these non-endogenous isoforms of APP, which express exons 7 and 8, as opposed to only exon 15, had previously been demonstrated to be up-regulated in AD brain [117,118,119]. Basing their hypothesis on the work of Makayev et al., the authors then investigated the influence of miR-124 on APP mRNA splicing through PTBP1. They showed that mouse neuronal Neuro2a cells that were treated with a synthetic miR-124 precursor had decreased expression of PTBP1 and of non-neuronal APP isoforms containing exons 7 and 8 as compared to scramble and mock transfection controls. Smith et al. also showed that Neuro2a cells that were treated with PTBP1 siRNA had decreased levels of non-neuronal APP isoforms as compared to scramble siRNA control, which suggests that miRNA regulates APP mRNA splicing through PTBP1. Most importantly, the authors found a significant decrease in miR-124 in human AD anterior temporal cortex as compared to control. These results suggest that down-regulation of miR-124 in AD brain causes up-regulation of PTBP1 and thus increased expression of non-neuronal isoforms of APP, which may be pathogenic due to their increased expression in AD.

BACE1 was identified as a possible target of miR-124 by Fang et al. in 2012 [120]. The authors showed that expression of BACE1 protein significantly increased in PC12 cells transfected with miR-124 inhibitor as compared to untransfected control, and that cell death increased under this condition, as well. BACE1 mRNA was also found to be a potential target for miR-124 via two prediction algorithms. Interestingly, the authors reported reduced levels of miR-124 in PC12 cells treated with Aβ as compared to untreated control, which suggests that Aβ may be both a cause and effect of down-regulation of miR-124. The results of this study are correlative, so additional research should be done before BACE1 is considered to be a definite target of miR-124.

**Figure 2:**
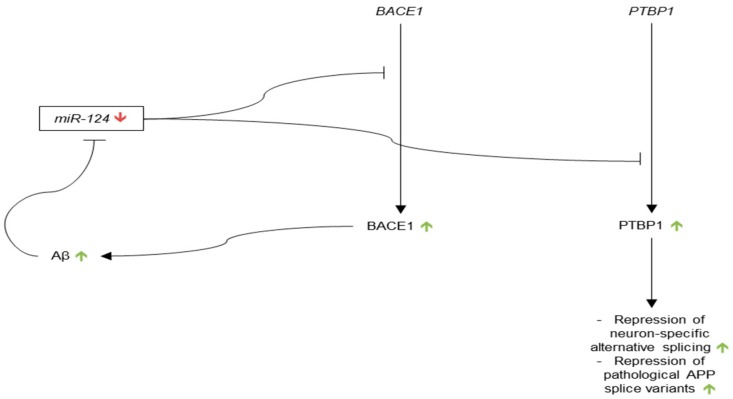
Regulation by microRNA (miR)-124. miR-124 inhibits the translation of BACE1 and Polypyrimidine Tract Binding Protein 1 (PTBP1). The down-regulation of miR-124 in AD brain is indicated by the red arrow. The hypothetical up-regulations of the target proteins and their cellular effects are denoted by the green arrows.

Most recently, miR-124 was found to be significantly reduced in the hippocampus of late-onset AD (LOAD) patients as compared to normal age-matched controls, thus providing additional evidence that miR-124 is in fact dysregulated in AD [121]. Future research into miR-124 may benefit from identifying additional target transcripts of PTBP1 that require neuron-specific splicing, as well as validating BACE1 as a target of miR-124 (Figure 2).

#### 3.3.3. microRNA-181

As early as 2004, Chen et al. reported significantly higher expression of miR-181 in mouse brain as compared to several other mouse tissues, suggesting a regulatory role for miR-181 in the CNS [122]. Several studies in 2007 and 2008 examined the role of miR-181 in the brain more closely. Schipper et al. found a 1.4-fold increase in miR-181b expression in the blood mononuclear cells (BMCs) of AD patients as compared to those of normal elderly controls and used prediction algorithms to show that miR-181b has putative binding sites on many of the down-regulated mRNAs in AD BMCs [123]. Additionally, Schipper et al. profiled the mRNA transcripts that were targeted by miR-181b and found that they were mostly genes involved in the cell cycle and DNA damage repair.

Two 2008 studies found miR-181 to be down-regulated in different areas of AD CNS. Cogswell et al. found miR-181a and -181c to both be down-regulated in the CSF of Braak V AD patients when compared to Braak I control [96]. miR-181a, b, c, and d were all observed to be down-regulated in the cerebellum and -181b and d were found to be down-regulated in the hippocampus. Similarly, Hebert et al. found miR-181c to be down-regulated in the cortex of sporadic AD brain as compared to control [97].

In 2010, Nunez-Iglesias et al. provided additional evidence of the down-regulation of miR-181 in AD brain by showing a significant decrease in the expression of miR-181c in the parietal lobe cortex of AD patients as compared to age-matched controls [124]. In the same year, Schonrock et al. reported on the results two studies that linked miR-181 down-regulation to increased expression of Aβ42 in mouse models of AD [99]. The authors showed a decrease in miR-181c expression in cultured mouse primary hippocampal neurons treated with Aβ42 as compared to phosphate-buffered saline (PBS) control. A decrease in miR-181c expression in the hippocampus in seven-month-old APP23 mice as compared to WT control was also observed. Additionally, Schonrock et al. showed that Aβ42 rapidly diminished the levels of miR-181c in the culture mouse neurons within 15 h, and, using prediction algorithm software, reported on several genes and pathways targeted by miR-181c, of which the MAPK signalling pathway is most affected.

In 2011, Wang et al. indicated that miR-181a and b were down-regulated in the temporal cortex of AD brain and that miR-181a and b were more highly expressed in temporal cortex white matter as opposed to grey matter.

The relationship between miR-181 and Aβ was further investigated by Geekiyanage et al. later in 2011 [125]. Using prediction algorithms, the authors found a putative binding site for miR-181c on the 3′-UTR of Serine Palmitoyltransferase Long Chain Base Subunit 1 (*SPTLC1*). The authors postulated that down-regulation of miR-181c may lead to increased *SPTLC1*, Serine palmitoyltransferase (SPT), ceramide, and thus increased levels of Aβ. To test this hypothesis, Geekiyanage et al. attached the 3′-UTR of human SPTLC1 to a luciferase construct and cotransfected it with either sense or antisense miR-181c in WT rat primary astrocytes. The authors found significant reductions in the levels of both SPTLC1 and ceramide as compared to scramble small interfering RNA (siRNA) control upon transfection with sense miR-181c. Furthermore, the levels of SPTLC1 and ceramide significantly increased in comparison to scramble siRNA control upon transfection with antisense miR-181c. The authors also showed a significant negative correlation between levels of miR-181c and SPTLC1 in the frontal cortices of control and AD patient brains. Most importantly, the authors showed that SPTLC1 expression was positively correlated with Aβ expression in AD brain, and that miR-181c lowered endogenous levels of SPTLC1 and Aβ in mouse primary astrocytes expressing the human APP Swedish mutation. The findings of Geekiyanage et al. thus complicated the understanding of the relationship between mir-181c and Aβ by suggesting that the down-regulation of miR-181c causes increased levels of pathogenic Aβ through dysregulation of SPTLC1. This finding, in combination with the 2010 Schonrock et al. study suggests that miR-181c may be involved in the production of Aβ through a positive feedback mechanism.

Since the publication of the 2011 study by Geekiyanage et al., much of the research into the role of miR-181 in AD has focused on its use as a biomarker of disease and on other potential target mRNAs. In reference to the use of miR-181 as a biomarker, a 2012 study by Geekiyanage et al. showed that expression levels of miR-181c are significantly decreased in the blood serum of AD and amnestic mild cognitive impairment/probable early AD patients as compared to that of control patients [110]. A 2013 study by Tan et al. found similar results using a larger cohort of AD patients [111].

**Figure 3:**
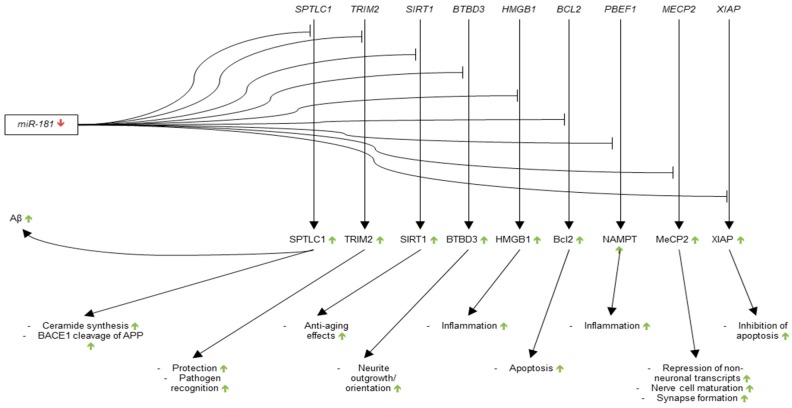
Regulation by miR-181. miR-181 inhibits the translation of Serine Palmitoyltransferase Long Chain Base Subunit 1 (SPTLC1), tripartite motif-containing 2 (TRIM2), silent mating type information regulation 2 homolog 1 (SIRT1), BTB Domain Containing 3 (BTBD3), high-mobility group protein 1 (HMGB1), B-cell lymphoma 2 (Bcl2), Nicotinamide phosphoribosyltransferase (NAMPT), methyl CpG binding protein 2 (MeCP2), and X-linked inhibitor of apoptosis (XIAP). The decreased levels of miR-181 in AD brain are denoted by the red arrow. The green arrows denote the hypothetical upregulation of the targeted proteins and their cellular effects.

As for the research into additional targets of miR-181, a 2012 study by Schonrock et al. showed significant decreases in the expression of TRIM2, SIRT1, and BTB Domain Containing 3 (BTBD3) in HeLa cells transfected with miR-181c as compared to control transfections [104]. A 2013 study by Hutchison et al. showed that high mobility group protein 1 (HMGB1), B-cell lymphoma 2 (Bcl-2), and nicotinamide phosphoribosyltransferase (NAMPT) that is associated with a biotinylated miR-181c probe in a pulldown assay and that Methyl-CpG binding protein 2 (MeCP2) and X-linked inhibitor of apoptosis (XIAP) reporter constructs were down-regulated by miR-181c in 293 cells as compared to firefly controls [126]. Contradictory to the findings of Schonrock et al., SIRT1 was not found to be regulated by miR-181c. Hutchison et al. also showed that down-regulation of miR-181b and c led to increased levels of pro-inflammatory cytokines in control and lipopolysaccharide-treated mouse primary astrocytes. These findings suggest that the miR-181 family has a wide range of regulatory roles in pathways potentially involved with AD pathogenesis (Figure 3).

#### 3.3.4. microRNA-29

Prior to 2008, miR-29 was primarily investigated in relation to cancer. miR-29 had been observed to be more highly expressed in astrocytes than in neurons in 2005, but besides this finding, AD-relevant research on miR-29 was limited [127].

In addition to one study in which miR-29a, b and c were found to be highly and uniformly expressed throughout 13 regions of mouse CNS, and thus likely an important regulatory miRNA in the brain [93], two articles were published in 2008 that reported different trends in dysregulation of miR-29 in AD. Cogswell et al. reported significant up-regulation of miR-29a and b in the medial frontal gyrus of Braak V-VI patients as compared to Braak 0-I control [96]. No change was observed, however, in the levels of miR-29a or b in Braak III-IV brain as compared to control. On the contrary, Hebert et al. reported down-regulation of miR-29b-1 in sporadic AD cortex as compared to age-matched control cortex [97]. miR-29b-1 and miR-29a were also predicted to target *BACE1* by several prediction algorithms. Having found significant inverse correlations between miR-29b-1 and miR-29a expression with BACE1 protein expression in the human cortex and cerebellum, the authors verified miR-29b-1 and miR-29a regulation of *BACE1* by showing reductions in luciferase activity in HeLa cells cotransfected with either miR-29b-1 or miR-29a, in addition to a luciferase construct containing the 3′-UTR of *BACE1*, as compared to scramble transfection control. miR-29b-1 and miR-29a were also shown to modulate BACE1 activity, as quantified by levels of Aβ and APP carboxy terminal fragment (CTF)-β, in HEK-293-APP-Sw cells.

The report by Hebert et al. indicating down-regulation of miR-29 was consistent with several subsequent publications. In 2009, down-regulation of miR-29a, b and c was observed in the cerebral cortex of the APPswe/PSΔE9 mouse model of AD in comparison to age-matched controls [128]. Additional in silico evidence of *BACE1* regulation by miR-29 was provided by Bettens et al. [129]. The authors also showed that genetic variability in the miRNA binding domains in the 3′-UTR of *BACE1* and in the mRNA binding regions of miR-29 did not significantly increase risk of AD, suggesting that *BACE1* is not overexpressed in AD due to an inability to bind miR-29, but more likely because miR-29 is down-regulated. This finding supports the hypothesis put forward in the 2008 publication by Hebert et al.

Additional evidence of miR-29 down-regulation in AD was produced in 2010 by Nunez-Iglesias et al. and Shioya et al., who observed miR-29a and miR-29b down-regulation in human AD parietal lobe cortex as compared to age-matched control [124], and miR-29a down-regulation in human AD frontal cortex as compared to non-neuropathological controls [130], respectively. Using several prediction algorithms, Shioya et al. also found miR-29a to be likely targets the transcript for neuron navigator 3 (NAV3), a protein that is primarily expressed in the central and peripheral nervous system [131]. The function of NAV3 is unknown, though it shares sequence similarities to the human NAV2 and *Caenorhabditis elegans* unc-53, which are involved in axon guidance and neurite outgrowth [132,133]. Regulation of *NAV3* by miR-29a was confirmed via luciferase assays in HEK-293 cells. NAV3 mRNA was additionally observed to be up-regulated in AD frontal cortex, though NAV3 protein was not, which is unusual. Although the authors hypothesized that NAV3 overexpression may be connected to neurofibrillary tangle formation, the role of NAV3 in AD has yet to be described.

In 2011, miR-29a, b and c were found to be enriched in human cerebral cortex grey matter as compared to white matter, and miR-29a and c were significantly correlated with the density of diffuse plaques in human AD cerebral cortex grey matter [100]. miR-29b was most closely correlated with density of diffuse plaques in human AD cerebral cortex grey matter, as opposed to density of neuritic plaques and neurofibrillary tangles in that region, though the correlation was slightly outside of the 95% confidence interval.

Three subsequent publications in 2011 discovered additional genes targeted by miR-29 that further illuminated the microRNA’s role in AD pathogenesis. First, Kole et al. found that miR-29b suppressed the expression of Bim, Bmf, Hrk, Puma, and N-Bak [134]. These five proteins, which are members of the Bcl-2 Homology 3( BH3)-only protein family, a group of proteins that enact the release of cytochrome c (cyt c) from the inner mitochondrial membrane into the cytoplasm in response to c-Jun phosphorylation during apoptosis [135], were shown to be suppressed by miR-29b via luciferase assay in HEK293T cells. The fact that miR-29b targets multiple members of the BH3-only family is significant because many of the BH3-only proteins have been shown to have redundant activities, meaning suppression of just one member is not sufficient to inhibit release of cytochrome c and halt apoptosis [136,137,138].

The capacity of miR-29b to suppress five of the eight known members of the BH3-only family suggests that miR-29b is capable of independently blocking apoptosis. Indeed, the rates of cell death in P3 sympathetic neurons that were exposed to nerve growth factor (NGF) deprivation, etoposide, or tunicamycin were drastically reduced in cells microinjected with miR-29b as opposed to those microinjected with control miRNA. Furthermore, under NGF deprivation conditions, cyt c was observed via immunofluorescence staining to be localized to the inner mitochondrial membrane of P5 neurons microinjected with miR-29b, whereas cyt c only faintly appeared in uninjected and control-miRNA treated neurons, a staining pattern that is typical of cyt c release into the cytoplasm. When considering that miR-29 has repeatedly been shown to be down-regulated in AD, it may be a key factor in the increased rate of neuronal apoptosis in AD and a particularly important target for future research.

Next, Zong et al. complemented the work of Hebert et al. by showing that *BACE1* is targeted by miR-29c, in addition to being targeted by miR-29a and b-1 [139]. The authors observed a negative correlation between levels miR-29c and BACE1 expression in SH-SY5Y cells, and showed that BACE1 protein expression decreased in HEK-293 cells that were transfected with miR-29c as compared to cells with control transfections. Direct interaction between miR-29c and *BACE1* was also demonstrated via luciferase assay. Importantly, miR-29c overexpression was shown to reduce the levels Aβ peptide in the brains of AD-like transgenic mice as compared to wild type controls.

Finally, SPTLC2 was identified as a target of miR-29a and b-1 by Geekiyanage et al. [125]. SPTLC2 is an essential protein in the synthesis of ceramides, which are lipids that have been found to be up-regulated in AD [140,141] and are believed to be involved in membrane transport of BACE1 via lipid rafts, thus causing increased APP cleavage and Aβ production [142,143]. miR-29a and b-1 were predicted to target *SPTLC2*. This interaction was confirmed via luciferase assay. The authors additionally demonstrated SPTLC2 and ceramide suppression in primary rat astrocytes that were transfected with either miR-29a or b-1, and observed significant negative correlations between SPTLC2 expression and levels of miR-29a and miR-29b-1 in human sporadic AD frontal cortices.

**Figure 4:**
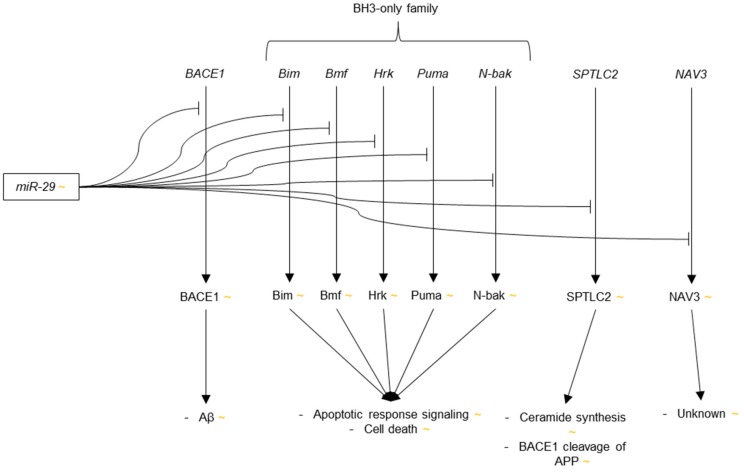
Regulation by miR-29. miR-29 inhibits the translation of BACE1, several Bcl-2 Homology 3 (BH3)-only family proteins (Bim, Bmf, Hrk, Puma, and N-bak), SPTLC2 and neuron navigator 3 (NAV3). The debatable levels of miR-29 expression in AD brain are indicated by the yellow tilde symbol (~), as are the hypothetical expression levels of the proteins regulated by miR-29 and their cellular effects.

Since 2011, research into miR-29 dysregulation in AD has mainly focused on the miRNA’s potential role as a biomarker for AD in human blood serum, plasma and CSF [110,112]. The current understanding of miR-29’s role in AD brain is represented in Figure 4.

#### 3.3.5. microRNA-34

miR-34 expression in both human and animal normal and AD brain and blood was described in a number of studies published in 2007 and 2008. First, human microRNA microarray profiling indicated significantly increased expression of miR-34a in the blood mononuclear cells of AD patients as compared to normal elderly controls [123]. This observation was validated by quantitative real-time polymerase chain reaction (qRT-PCR) measurements. miR-34a was also predicted by miRBase to target numerous AD-relevant transcripts, though none of the predicted targets were validated. Significant up-regulation of miR-34a, as well miR-34b and c, in Braak III, IV, V, and VI cerebellum, hippocampus, and medial frontal gyrus, as compared to Braak 0 to I samples, was subsequently observed by Cogswell et al. [96].

These results, which indicate consistent up-regulation of miR-34 in AD, were concurrently published with two studies that further described miR-34 activity in the CNS. In a study by Bak et al., RT-PCR was employed to show that levels of miR-34a were six to nine-fold higher in mouse medulla oblongata, pons, and spinal cord than in the rest of the mouse brain [93]. In a study by Parsons et al., the levels of miR-34a and c were measured in the hippocampus of several inbred mouse strains [144]. Of importance to AD, miR-34c was correlated with learning and memory measures for the mice.

Following this series of publications profiling miR-34 in normal and AD brain, Wang et al. identified the first AD-relevant target of miR-34a in 2009 [128]. To reconfirm the previous results on miR-34 up-regulation in AD, levels of miR-34a were measured in the cerebral cortex of three and six-month-old APPswe/PSΔE9 and compared to age-matched controls. Consistent up-regulation of miR-34a in the APPswe/PSΔE9 mice was observed. Bcl2, an antiapoptic protein responsible for inhibition of caspase-9 and neuroprotection from NFTs and Aβ [145,146], was predicted as a target of miR-34a via Targetscan, and *BCL2* regulation by miR-34a was validated via luciferase assay in 293T cells. Accordingly, Bcl2 was found to be down-regulated in the same APPswe/PSΔE9 mice as compared to age-matched controls, while caspase-3 was found to be up-regulated. The increased levels of miR-34a in AD may thus decrease the expression of Bcl2 and make neurons more susceptible to apoptosis.

miR-34c activity in mouse hippocampus was further described by Zovoilis et al. in 2011 [147]. miR-34c was found to be enriched in mouse hippocampus as compared to whole brain tissue. Knowing that miR-34a had been shown to target the transcript for SIRT1 in human colon cancer cells [148], the authors hypothesized that the previously reported up-regulation of miR-34c in AD may lead to decreased production of SIRT1 in the hippocampus, and thus diminished performance in memory-based tasks due to the fact that SIRT1 production in the hippocampus had been correlated with memory formation and learning in mice. Indeed, in two mouse models of AD: 24-month-old C57B1/6J mice and APPPS1-21 mice, miR-34c was found to be up-regulated, while SIRT1 was down-regulated. These findings were supported by a luciferase assay showing that miR-34c targets *SIRT1* in neurons. Most importantly, the inferior performances of 24-month-old C57B1/6J mice and APPPS1-21 mice in fear conditioning memory tests as compared to controls that were abrogated by both *SIRT1* binding site protectors and miR-34c seed inhibitors, as were SIRT1 protein levels in the hippocampus. This study thus provides compelling evidence of miR-34c suppression of memory formation through targeting of SIRT1 mRNA.

In 2012, Liu et al. provided additional information on the role of miR-34 in aging [149]. The expression of miR-34c, though not that of miR-34a or b, was observed to increase with age in drosophila brain, which was unique among all of the miRNAs detected. To probe the function of miR-34c in drosophila brain, miR-34^−/−^ mutant flies were generated. The miR-34^−/−^ flies had decreased lifespan, diminished performance in locomotion and stress-resistance tests, accelerated production of age-related vacuoles in the brain, and increased the transcription of genes that were associated with aging. Prediction algorithms indicated that miR-34 may target *Ecdysone-induced protein 74EF* (*Eip74EF*), which encodes E74A, a component of steroid signalling pathways implicated in the regulation of lifespan. E74A expression was observed to be negatively correlated with the levels of miR-34 in drosophila brain. Finally, miR-34^−/−^ flies with reduced expression of E74A had increased lifespan, and decreased vacuole production, pathogenic polyQ protein inclusion, and neural degeneration as compared to miR-34^−/−^ flies without altered expression of E74A. Up-regulation of miR-34 was also observed to increase lifespan as compared to control drosophila, which led the authors to suggest that up-regulation of miR-34 may have anti-neurodegenerative effects. Although these results may appear to be incongruous with previous literature on miR-34 dysregulation in AD, it is important to note that these results were obtained in drosophila, and that E74A was not validated as a target of miR-34 via luciferase assay. Additionally, miRNAs have hundreds of targets, so miRNA knockout models may have many problems not observed in models of miRNA dysregulation. Furthermore, evidence of lifespan extension in miR-34 knockout *C. elegans* was published in the following year, so these results are controversial [150].

In 2013, tau mRNA was shown to be a target of miR-34a via luciferase assay in M17D and HEK293 cells [151]. As the authors note, however, both miR-34a and tau are overexpressed in AD, so the effect of miR-34a regulation on expression of tau is likely insignificant in comparison to the effects of other pathological mechanisms, leading to increased production of tau.

Most recently, levels of miR-34a in AD plasma and CSF were observed to be reduced as compared to healthy controls, suggesting a possible use for miR-34a as a non-invasive biomarker for AD [112].

**Figure 5:**
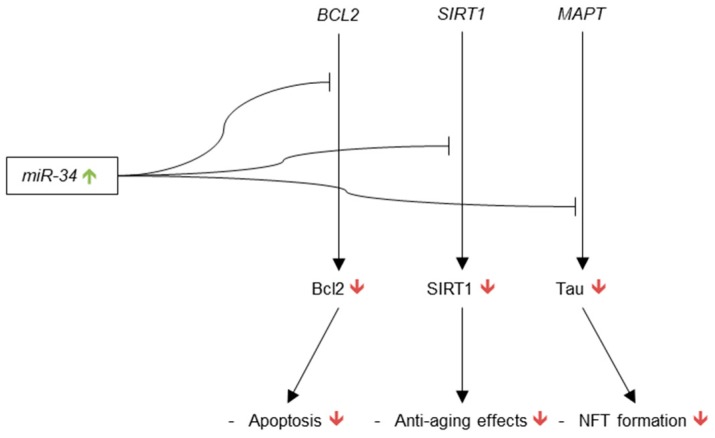
Regulation by miR-34. miR-34 inhibits the translation of BCL2, SIRT1, and Tau. The green arrow indicates the increased expression levels of miR-34 in AD brain. The red arrows indicate the hypothetically decreased levels of the targeted proteins and their cellular effects.

It is important to note that a significant body of cancer research has shown that tumor protein p53 promotes the transcription of miR-34 [152]. These studies have not been conducted in cellular or animal models of AD so the implications for Alzheimer’s research are tentative. Aβ42, however, has been shown to activate the p53 promoter and induce p53 expression in SK-N-SH cells [153], so evidence of p53 regulation of miR-34 in AD could reveal an important pathogenic system in AD and explain the possible root cause of the increased expression of miR-34 in AD brain. The current understanding of miR-34’s role in AD brain is represented in Figure 5.

#### 3.3.6. microRNA-107

miR-107 was reported to be specifically down-regulated in the temporal cortex during the early stages of AD, and to be correlated with the up-regulation BACE1 by Wang et al. in 2008 [154]. The authors observed a continual decrease in the expression of miR-107 in the temporal cortex from non-demented, without pathology patients to non-demented, with pathology patients to MCI patients, to AD patients. Due to the significant reductions in miR-107 expression in MCI brain as compared to non-demented, without pathology brain, the authors postulated that miR-107 down-regulation occurs in the earliest stages of AD. In the same article, miR-107 was predicted to target several miRNA recognition elements (MREs) on the *BACE1* 3′-UTR. The authors found a significant negative correlation between miR-107 expression and BACE1 protein levels in the temporal cortex brain samples. miR-107 reduced the levels of luciferase reporter constructs containing the *BACE1* 3′-UTR, an effect that was abrogated by mutation of the *BACE1* MREs. In order to verify these findings on BACE1 regulation by miR-107, Nelson and Wang published a validation study in 2010 that showed negative correlations between miR-107 expression and *BACE1* mRNA levels, as well as miR-107 expression and neuritic plaque counts in human temporal cortex samples [155].

In 2010, two additional AD-related targets of miR-107 were identified: granulin (GRN), which is a neurotrophic factor involved in neurite outgrowth [156], and cofilin, an actin-binding protein responsible for actin-filament disassembly. Granulin, which has been reported to be down-regulated in AD [157], was found to be the mRNA most strongly targeted by miR-107 in H4 cells [158]. The authors identified the 5′ seed sequence of miR-107 that binds to *GRN*, as well as the corollary seed sequence, which was found to be located in the open reading frame of *GRN*. These findings were confirmed by demonstrating significant reductions in *GRN* expression in H4 cells that were transfected with miR-107 as compared to control miRNA transfection, as well as in H4 cells treated with glucose, a known up-regulator of miR-107 [159], as compared to untreated controls. Interestingly, after depth-controlled cortical impact brain injury in mice, concurrent down-regulation of miR-107, and up-regulation of GRN in hippocampal CA1 and CA3 was observed on the side of brain ipsilateral to the insult, as compared to that on the contralateral side of the impact. The authors suggested that this model of traumatic brain injury resembles AD pathology, and may be analogous to miR-107 and GRN dysregulation in AD. Granulin, however, has previously been demonstrated to be down-regulated in AD brain [157], so this model may be inaccurate.

Cofilin 1 (CF1), which is one of the main components of the rod-shaped Hirano bodies that are found in AD brain [160], was identified as a target of miR-107 by Yao et al. [161]. Concomitant up-regulation of CF1 and down-regulation of miR-107 was observed in APP transgenic mouse (Tg19959) brain as compared to WT brain. The fraction of neurons containing rod-like structures immunoreactive for CF1 was significantly higher in Tg19959 brain than in WT brain, as well. Observing that levels of CF1 mRNA were equivalent in Tg19959 and WT brain, the authors hypothesized that CF1 is likely a target for translational suppression by miR-107. Sequence similarities between miR-107 and the 3′-UTR of *CF1* were found using several prediction algorithms, and miR-107 was subsequently found to repress luciferase activity in HEK-293 cells co-transfected with miR-107 and a luciferase reporter construct containing the 3′-UTR of *CF1* as compared to oligo transfection control. Transfected anti-miR-107 inhibitor was additionally found to increase CF1 expression in Swe-N2a cells in comparison to oligo transfection control.

In 2011, in addition to being found to be enriched in human AD cortical grey matter in comparison to white matter and correlated with density of diffuse plaques in grey matter [100], miR-107 was reported to regulate another mRNA pertinent to AD: *CDK5R1* [162]. *CDK5R1* encodes p35, which is an activator of Cdk5, an enzyme that is involved in the development and function of the CNS and is essential for neuronal survival. Hyperactivation of Cdk5, due in part to the up-regulation of p35, is associated with AD [163]. miR-107 was predicted to bind *CDK5R1* by the prediction algorithms PicTar and RNAhybrid. A significant inverse correlation was also observed between miR-107 and p35 in SK-N-BE, SH-SY5Y, HEK-293, DU-145, and MCF-7 cells. The interaction between miR-107 and *CDK5R1* was confirmed by demonstrating down-regulation of p35 in SK-N-BE cells transfected with pre-miR-107 and up-regulation of p35 in SK-N-BE cells transfected with anti-miR-107. Decreased luciferase activity in SK-N-BE cells co-transfected with pre-miR-107 and a luciferase construct containing the 3′-UTR of *CDK5R1* was also detected. Additionally, the precise seed sequence on *CDK5R1* was located, and the transcript was found to be subject to translational repression as opposed to degradation. These results are consistent with previous findings showing that miR-107 is down-regulated in AD brain, whereas p35 and Cdk5 activity, are up-regulated in AD brain.

A Disintegrin and Metalloproteinase 10 (*ADAM10*), which encodes an α-secretase that cleaves within the Aβ domain of APP and generates the neurotrophic sAPPα, was identified as a target of miR-107 in 2012 by Augustin et al. [164]. The authors employed a number of prediction algorithms in a careful workflow to identify miR-107 as a regulator of *ADAM10*. This regulatory interaction was experimentally validated by showing a 52% reduction in relative light units in SH-SY5Y cells co-transfected with miR-107 and a luciferase construct containing the 3′-UTR of *ADAM10* as compared to mock transfection control.

*ADAM10* is atypical among miRNA targets in AD due to the fact that up-regulation of *ADAM10* has neurotrophic, anti-AD properties. Further examination is required to clarify the relationship between pathological down-regulation of miR-107 and the neurotrophic up-regulation of *ADAM10*.

Most recently, in 2014, miR-107 was observed to be down-regulated in the hippocampus of Braak VI patients as compared to Braak III-IV and normal controls, though it was undetectable in CSF [165]. miR-107 was also found to be regulated by soluble Aβ (sAβ) in 7PA2 cells through a mechanism that was elicited by oxidative stress [166].

**Figure 6:**
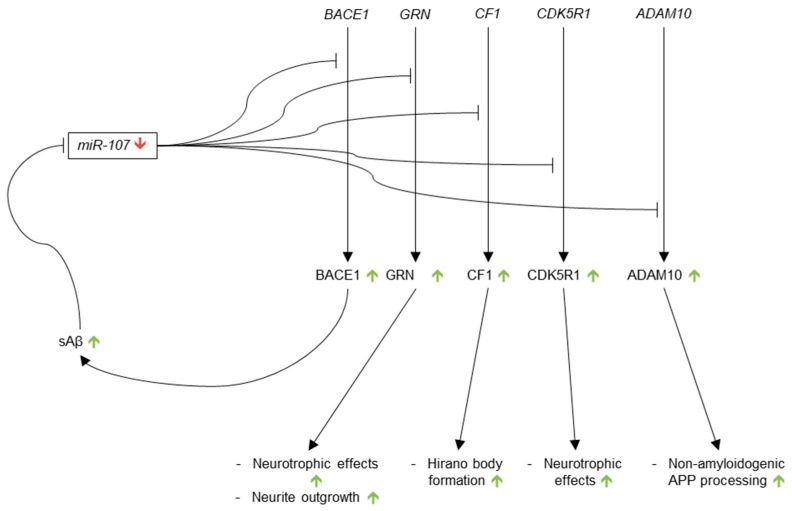
Regulation by miR-107. miR-107 inhibits the translation of BACE1, GRN, CF1, CDK5R1, and ADAM10. The red arrow indicates the downregulation of miR-107 observed in AD brain. The green arrows indicated the hypothetically increased levels of the proteins targeted by miR-107, as well as their cellular effects.

The current literature consistently demonstrates decreased levels of miR-107 in AD brain. Five AD-relevant targets of miR-107 have been identified, as well: *BACE1*, *GRN*, *cofilin*, *CDK5R1*, and *ADAM10* (Figure 6). If miR-107 is in fact an influential regulator of these five mRNAs, the proteins they encode should be up-regulated in AD. While up-regulation of BACE1, cofilin and p35 (encoded by *CDK5R1*) is consistent with AD pathology, increased levels of the WT forms of the neurotrophic factors GRN and ADAM10 are not observed in AD. Further investigation into these discrepancies is required.

#### 3.3.7. microRNA-146

In 2008, Cogswell et al. found miR-146b to be down-regulated in the cerebellum, hippocampus, medial frontal gyrus, and CSF of Braak III-IV AD patients as compared Braak I controls [96]. That same year, Lukiw et al. provided evidence to suggest that nuclear factor kappa-B (NF-κB) promotes the transcription of miR-146a, which in turn, suppresses translation of complement factor H (CFH) [167], which is a repressor of inflammatory responses in the brain. This system is dysregulated in AD and its dysregulation leads to increased neurodegeneration through excessive inflammatory response in the brain [168]. The authors reported increased levels of NF-κB and miR-146a in the neocortex and hippocampus of a cohort of AD patients as compared to a control cohort, as well as decreased levels of CFH in those same areas. Using human neural primary cell culture, the authors demonstrated that the expression of miR-146a increased in response to common causes of inflammation in AD, such as IL-1β and Aβ42 and H_2_O_2_. The increase in miR-146a was partially eliminated in the presence of NF-κB inhibitor PDTC, suggesting that miR-146a is up-regulated by NF-κB in response to cellular stress. The authors also showed that H_2_O_2_ reduced levels of CFH, and that co-transfection with anti-miR-146a siRNAs abolished this effect. Since CFH helps regulate immune and inflammatory responses in the brain, its down-regulation by miR-146a in response to inflammation suggests that miR-146a is involved in a pathogenic positive feedback loop that leads to increased susceptibility to and damage from causes of inflammation, such as Aβ42.

In 2009, Sethi and Lukiw followed up on Lukiw’s initial findings on miR-146a. The authors found that miR-146a expression in the temporal lobe neocortex of AD patients was significantly increased as compared to those in patients with ALS, Parkinson’s disease and Schizophrenia [98]. This finding suggests that the pathogenic system in which miR-146 dysregulation is involved may be specific to AD among neurological and neurodegerative disorders. Sethi and Lukiw also demonstrated that miR-146 has a half-life of approximately 90 min in cultured, primary human neural cells.

Contrary to Lukiw’s reports on miR-146a, Schonrock et al. published findings in 2010 that indicated slight down-regulation of miR-146a in cultured mouse primary hippocampal neurons that were exposed to Aβ42 as compared to those exposed to PBS control [99]. Additional evidence of the up-regulation of miR-146a in response to Aβ42 stress provided by Lukiw et al. in the following years, however, strongly suggests that miR-146a is in fact up-regulated in AD.

In 2011, Li et al. showed that miR-146a was up-regulated in cultured human neuronal-glial (HNG), human astroglial (HAG) and (HMG) cells treated with Aβ42 and TNFα as compared untreated controls [169]. Expanding upon Lukiw’s 2008 findings on CFH down-regulation in response to increased levels of miR-146a, Li et al. showed decreased mRNA and protein expression of CFH and Interleukin-1 receptor-associated kinase 1 (IRAK-1), an enzyme that contributes to the up-regulation of NF-κB, in stressed HNG, HAG and HMG cells. Additionally, the authors observed decreased expression of the mRNA and protein expression of TSPAN12, which has been indicated in non-amyloidogenic APP cleavage as a binding partner of ADAM10 [170], in stressed HNG and HAG cells as compared to untreated controls.

Later in 2011 and in 2012, two publications provided additional evidence of miR-146a regulation by NF-κB. First, Pogue et al. showed that HAG cells stressed with a neurotoxic combination of iron and aluminium sulfates had significantly increased levels of ROS, NF-κB, and miR-146a as compared to untreated controls [171]. Importantly, the authors showed that the increases miR-146a expression observed in stressed cells were abolished by treatment with three NF-κB inhibitors: curcumin, PDTC, and CAY10512.

To confirm these results in a model more similar to AD, Lukiw treated primary culture HNG cells with a neurotoxic combination of IL-1β, and Aβ42 and detected significant up-regulation of NF-κB and miR-146a as compared to untreated controls [172]. Significant increses in miR-146a were not detected in stressed cells that were treated with the NF-κB inhibitors PDTC or CAY10512. Lukiw also reported down-regulation of both CFH and TSPAN12 mRNA and protein in the stressed cells, two proteins whose down-regulations by miR-146a lead to increased neuroinflammation and amyloidogenesis, respectively.

The string of publications dealing with miR-146a dysregulation in AD provides a fairly detailed portrait of the role of miR-146a in AD pathogenesis. Causes of cellular stress, such as Aβ42, IL-1β, TNFα, and several ROS up-regulate NF-κB, which in turn, promotes transcription of miR-146a. Increased levels of miR-146a lead to suppression of transcripts encoding proteins such as CFH, IRAK-1 and TSPAN12. Down-regulation of these proteins can lead to increased inflammation and amyloidogenesis in the brain. It is important to note that the mRNAs for these proteins have not been shown to bind miR-146a. That is miR-146a has been shown to be involved in their regulation, but it has not been shown to directly bind CFH, IRAK-1, or TSPAN12.

**Figure 7:**
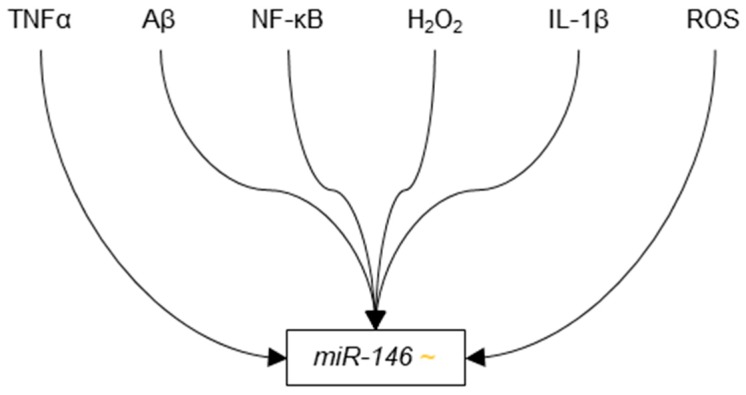
Regulation of miR-146. miR-146 expression is induced by multiple inflammatory species (Tumor necrosis factor (TNF)-α (TNFα), nuclear factor kappa-B (NF-κB), H_2_O_2_, interleukin (IL)-1β (IL-1β), and reactive oxygen species (ROS)), as well as Aβ. The yellow tilde (~) indicates the debated levels of miR-146 in AD brain.

Since Lukiw’s publication in 2012, miR-146a and b dysregulation has been tentatively associated with PS2 mutations in familial and sporadic AD (Jayadev et al., 2013). miR-146a has also been investigated as a potential AD biomarker in plasma and CSF [112]. The current understanding of miR-146’s role in AD brain is represented in Figure 7.

#### 3.3.8. microRNA-101

In 2008, Hebert et al. reported a decrease in miR-101 expression in the anterior temporal cortex and cerebellum of sporadic AD brain as compared to control [97]. Using several prediction algorithms, the authors also found that miR-101 had a candidate binding site on the 3′-UTR of *APP*.

These initial findings were followed by a number of articles published in 2010 that further explored the involvement of miR-101 in AD pathology. First, Nunez-Iglesias et al. reported on the results of a similar genome-wide miRNA profiling study, which found reduced miR-101 expression in the parietal lobe cortex of AD brain as compared to control [124].

Next, Vilardo et al. reported on a number of assays that provided evidence of an interaction between miR-101 and APP mRNA [173]. The authors showed significantly decreased expression of APP protein in rat hippocampal neurons upon the translational inhibition of the Ago2 component of RISC, demonstrating that APP translation is regulated by RISC, and thus by one or more miRNAs. The authors identified miR-101 as a likely regulator of *APP* translation using prediction algorithms. To directly assay the interaction between miR-101 and the 3′-UTR of *APP*, Vilardo et al. co-transfected PC12 cells with reporter constructs containing the luciferase CS joined to the 3′-UTR of APP with synthetic miR-101 precursor, and found that luciferase expression was significantly reduced as compared to firefly control. They also showed that site directed mutagenesis of the putative binding site of miR-101 in the 3′-UTR of APP eliminated this reduction. The authors confirmed this effect in vivo by showing that both APP protein, and, to a lesser extent, mRNA expression decreased significantly in rat hippocampal neurons that overexpressed miR-101. APP protein levels significantly increased in the same cells upon transfection with miR-101 hairpin inhibitors.

**Figure 8:**
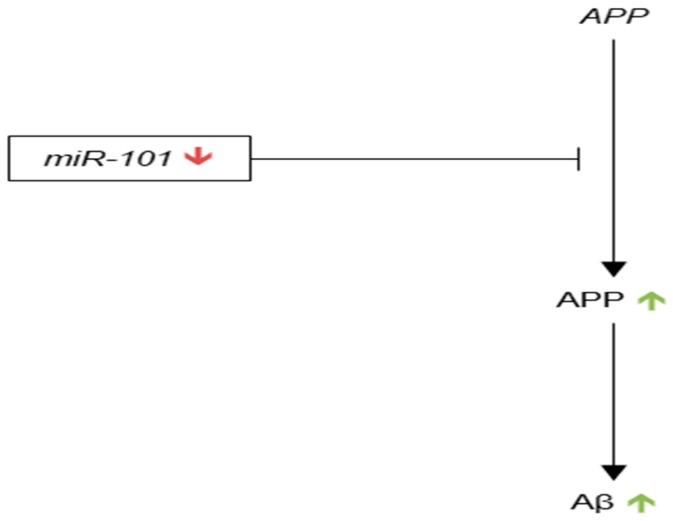
Regulation by miR-101. miR-101 inhibits the translation of APP. The red arrow indicates the significant downregulation of miR-101 expression observed in AD brain. The green arrows indicated the hypothetically increased levels of APP and Aβ resulting from the downregulation of miR-101.

Long et al. published a similar article later in 2011, which reconfirmed that miR-101 binds to a site in the 3′-UTR of *APP* [174]. Expanding upon the work of Vilardo et al., which was done in rat hippocampal neurons and the murine PC12 cell line, Long et al. reported significant reductions in APP expression in HeLa and human astroglial U373 cells upon transfection with miR-101 mimic as compared to control. The authors also compared miR-101 expression levels across seven cultured human cell types (HeLa, 293T, U373, NT2, Naïve SK-N-SH, Differentiated SK-N-SH, and NT2N) and found the highest expression levels in CNS-like differentiated NT2N neurons, which again suggests that miR-101 is an important regulator of APP translation in human CNS neurons. It is important to note that Long et al. did not find a significant reduction in APP mRNA levels in HeLa cells transfected with miR-101 mimic and, in contrast to Vilardo et al., contended that miR-101 does not affect APP mRNA degradation. Both groups agree, however, that miR-101 most likely works primarily through translational inhibition of APP (Figure 8).

#### 3.3.9. microRNA-195

Cogswell et al. first identified miR-195 dysregulation in the AD brain in 2008. The authors found a decrease in the expression of miR-195 in the CSF of Braak V patients as compared to its expression in Braak I control patients [96]. In 2011, Wang et al. reported similar results, and showed that miR-195 was enriched in the grey matter of the superior and middle temporal cerebral cortex as compared to the white matter of those regions in AD brains [100]. The authors also suggested miR-195 is down-regulated in AD brain, though they did not provide data. Importantly, Wang et al. found a negative correlation between miR-195 expression and diffuse plaque (DP) density in the temporal cerebral cortex grey matter. This finding suggested that miR-195 may participate in the regulation of Aβ and that the down regulation of miR-195 in AD brains may lead to increased levels of pathological Aβ plaques.

The implication of the negative correlation between miR-195 expression and the DP density found by Wang et al. was substantiated by Zhu et al. in 2012. The authors first identified two sites on the 3′-UTR of *BACE1* as potential targets for miR-195 [175]. Using a luciferase reporter construct with the 3′-UTR of *BACE1* in HEK293 cells, the authors showed that miR-195 reduced luciferase activity as compared to vector.

These results provided compelling evidence of an interaction between miR-195 and the 3′-UTR of *BACE1*, which the authors further evinced by showing significant reductions in the intensity of BACE1 Western blots in N2a/WT cells transfected with miR-195 as compared to control vector, as well as significant reductions in Aβ40 and Aβ42 levels in miR-195-transfected N2a/APP695 cells. The authors additionally showed that si-miR-195 increased the levels of BACE1, Aβ40, and Aβ42 as compared to si-control. It is important to note that the authors did not observe significant differences in BACE1 mRNA levels in the presence of vector, miR-195, si-control, or si-miR-195, which suggests that miR-195 may act by inhibiting translation of BACE1 mRNA as opposed to degrading BACE1 mRNA transcripts.

**Figure 9:**
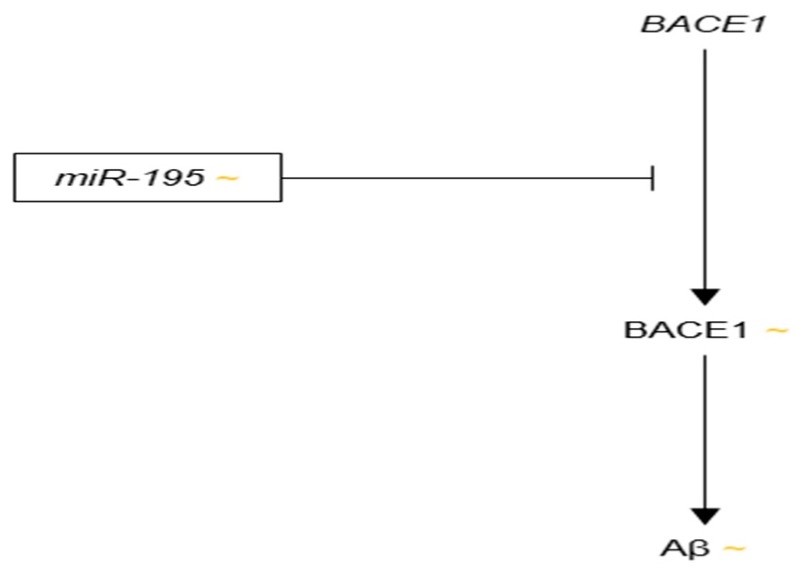
Regulation by miR-195. miR-195 inhibits the translation of BACE1. The yellow tildes (~) indicate the lack of consensus on the expression levels of miR-195 in AD brain, as well as the hypothetically unclear effects miR-195 on the levels of BACE1 and Aβ.

Though there is a relative lack of publications devoted to the characterization of miR-195 in AD, the few that have been written on the subject have provided a fairly cohesive portrait of the miRNA’s role in AD: miR-195 appears to be an inhibitor of BACE1 mRNA translation (Figure 9). Down-regulation of miR-195 in AD brain leads to increased expression of BACE1 protein, increased production Aβ40 and Aβ42, and finally increased formation of pathogenic amyloid plaques. A 2013 article that was published by Lau et al., however, did show an increase in the levels of miR-195-5p in the hippocampus of LOAD patients as compared to control, so further investigation of miR-195 expression in AD brain is required [121].

#### 3.3.10. microRNA-153

The involvement of miR-153 in neurodegenerative disorders was first detected in relation to α-synuclein regulation in PD, a protein that is also involved in familial and sporadic AD pathology. In 2009, Junn et al. briefly reported on the results of a prediction algorithm study that detected a putative miR-153 binding site in the 3′-UTR of α-synuclein, suggesting that the down-regulation of miR-153 may contribute to formation of excess α-synuclein and Lewy bodies observed in AD brain [176].

Doxakis identified the same binding site using similar prediction algorithms in 2010 and provided direct evidence of α-synuclein regulation by miR-153 [177]. Using a reporter construct containing the *α-synuclein* 3′-UTR in HEK293 cells, Doxakis showed that miR-153 produced a 12% decrease in luciferase activity, an effect that was eliminated by point mutations on the binding site. Demonstrating the interaction in another way, Doxakis showed that the removal of the 3′-UTR of α-synuclein from the reporter construct abolished the inhibitory effect of miR-153. These results were corroborated in cortical neurons. Doxakis also noted that α-synuclein mRNA levels were reduced by 37% as compared to control in HEK293 cells co-transfected with the full α-synuclein plasmid and miR-153 expression plasmids, suggesting that miR-153 regulates α-synuclein expression at least in part by the degradation of α-synuclein mRNA. Finally, Doxakis surveyed miR-153 expression in several tissues of postnatal day 1 mice and found that miR-153 was most highly expressed in neural tissues, such as the midbrain, hippocampus, and cortex, and that within neural tissue samples miR-153 was more highly expressed in neurons as opposed to astrocytes.

**Figure 10:**
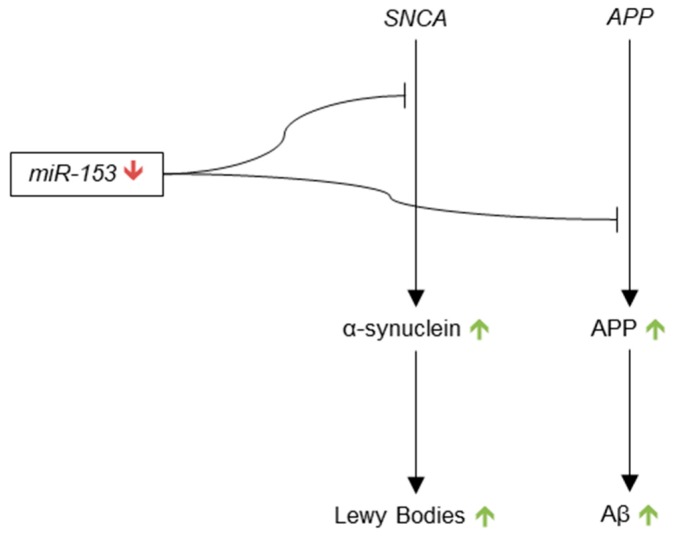
Regulation by miR-153. miR-153 inhibits the translation of α-synuclein and amyloid precursor protein (APP). The red arrow indicates that miR-153 expression is decreased in AD brain. The green arrows denote the hypothetically increased levels of α-synuclein, Lewy bodies, APP, and Aβ resulting from miR-153 downregulation.

In 2012, Long et al. augmented the understanding of miR-153 regulation in neurodegerative disease by providing evidence of miR-153 dysregulation in AD brain and of an interaction between miR-153 and APP [178]. Using methods that were similar to those of Doxakis, Long et al. discovered a putative binding site for miR-153 on the 3′-UTR of APP. The authors first showed APP protein and mRNA levels were significantly reduced in HeLa cells transfected with miR-153 mimic. They also demonstrated that miR-153 inhibitor increased APP expression in human fetal brain culture as compared to mock transfection control. Importantly, the authors found that miR-153 significantly reduced the expression of Aβ40 and APLP2 in human fetal brain, and that miR-153 expression decreases with increasing Braak stage. The current understanding of miR-153’s role in AD brain is represented in Figure 10.

#### 3.3.11. microRNA-132/212

miR-132 has strong modulatory effects in the CNS. In a recent study, Hansen et al. [179] employed a combination of transgenic mouse models and conditional knockout to investigate the contribution of miR-132/-212 gene locus on learning and memory, before assessing the unique effects of each microRNA on gene expression levels in the hippocampus. They found that miR-132/-212 double-knockout mice showed significant cognitive deficits in recognition and spatial memories, as well as in tests involving novel object recognition. A Further study discovered significant enrichment of the genes that are related to neuronal proliferation, synaptic transmission and morphogenesis, all of which are well-known for their function in learning and memory formation thus strongly confirming the role of miR-132/-212 gene locus as a key regulator of cognitive capacity. They hypothesized that the dysregulation of miR-132/-212 expression may contribute to signalling mechanisms implicated in a vast number of cognitive disorders.

Although miR-132/212 is the only miRNA that is consistently downregulated in different brain areas, it is inversely correlated with Braak stage [180]. It was reported that miR-132/212 deficiency in mice leads to increased tau phosphorylation, aggregation, and expression. Nevertheless, the treatment of AD mice with miR-132 restores in part, tau metabolism and memory function. miR-132 level is also correlated with insoluble tau as well as cognitive impairment in humans, indicating the important role of miR-132/212 in regulating tau pathology in both mice and humans, thereby providing new alternatives for the development of new therapeutic agents.

In a recent study, it was reported that miR-132/212 was down-regulated in the frontal cortex of subjects with mild cognitive decline [181] (Weinberg 2015). Taken into consideration the role of frontal cortex in responding to the onset of dementia via neuronal reorganization, their data suggest that miRNA-mediated up-regulation of the sirt1 pathway represents a compensatory response to the onset of dementia, which indicates some therapeutic potential of the target in future.

## 4. Therapeutic Targets for miRNAs

RNA interference (RNAi) based therapies are becoming a major class of potential therapeutics due to their success in knocking down the expression of selected targeted genes and they offer alternate treatment options when the prevailing drug technology is not satisfactory [182]. Endogenous RNAi was described in 1998 by Andrew Fire, Craig Mello et al. [183]. From a therapeutic perspective, RNAi involves the delivery of small RNA duplexes, including miRNA mimics, siRNAs, short hairpin RNAs (shRNAs), and Dicer substrate RNAs (dsiRNAs) [184]. miRNAs are gaining attention as useful therapeutic targets because of their role in regulating endogenous gene expression [185]. Currently, two types of miRNA targets are being explored: miRNA mimics and anti-miRNAs. The miRNA mimics resemble miRNA precursors and are helpful in down-regulating the expressions of directly targeted proteins or gene-related proteins contributing to AD pathogenesis. Therefore, reducing specific protein levels that are involved in AD pathogenesis can be a protective therapeutic strategy.

The process of miRNA generation (described above), which involves the initial synthesis of the long “primary” miRNA in the nucleus, its subsequent translocation to the cytoplasm, the “gating” of pre-miRNA precursor via the nuclear pore, the pre-miRNA itself, and finally, the formation of mature miRNA constitute potentially important therapeutic targets in anti-miRNA targeted therapies [80]. The aim of anti-miRNA therapies is to either completely or partially vanish the function of the miRNA of interest [186]. In situations in which miRNAs are over-expressed, a complementary RNA sequence is injected in order to inactivate miRNAs by preventing translation. In 2005, Krutzfeldt et al. reported the design of RNA snippets that were conjugated to cholesterol molecules (antagomirs) that help RNA to enter cells [187]. However, antagomirs cannot cross blood brain barrier, though they are able to penetrate brain cells if directly injected into the brain [188]. Further research is warranted in order to determine the exact efficacy and toxicity of antagomirs. Another key strategy to ensure successful implementation of anti-miRNA therapies is personalized treatment approaches aimed at identifying specific miRNA deficits in individual AD patients [80].

The miRNA-targeting locked nucleic acids (LNAs) are analogues of RNA that can bind to complementary RNA [189]. LNA-modified anti-miRNAs hinder miRNA binding to its targets and reduce miRNA levels. However, investigations on the effectiveness of LNAs need to be tested in models of neurodegeneration.

Using miRNAs as therapeutic targets may provide an important breakthrough in neurodegenerative disease therapies if appropriately understood and safely manipulated. The miRNAs discussed in this review have differential expression in AD and appear to potentially fulfill multiple regulatory roles in multiple pathways involved in AD pathology (Table 1). Even though miRNA research has been a significant area of investigation in cancer for a number of years, it is still a relatively new area for research in AD.

Although we have included some papers describing on northern blots as a method of measuring miRNA expression, we would like to caution that the method is time-consuming, requiring large amounts of sample input, and are therefore unsuitable for high-throughput screens [190]. Some of the utilised methods have to be more specific, and therefore one must be cautious about extrapolating the data from some studies when conducting similar testing in animals or cell culture.

## 5. Conclusions

It is clear that much work remains to be accomplished to elucidate and define the various roles of multiple miRNAs in pathological pathways due to multiple miRNA-target interactions. Due to their wide array of mRNA targets, miRNAs are able to regulate various cellular programmes.

Several studies have investigated drugs that can target dysregulated miRNAs in AD and affect reductions in AD pathology either in vitro or in vivo. Research into miRNA-targeting drugs may be the logical next step in AD-dysregulated miRNA research. Additionally, reviewing a number of miRNAs that demonstrate evidence of involvement in AD indicates that other than amyloidogenic and inflammatory pathways, potential regulation of other pathways that are involved in AD by these molecules has been largely ignored. Therefore, the investigation of alternative pathways and mechanisms, in addition to research into miRNA-targeting drugs, is warranted and holds potential for the development of novel therapies for the treatment of AD.

## Figures and Tables

**Table 1:** microRNA Expression Profiles, Targets, and Regulators.

microRNA	Evidence of Up-Regulation in AD Brain	Evidence of Down-Regulation in AD Brain	AD-Relevant Targets	AD-Relevant Regulators
**miR-9**	− ↑ AD human CA1 hippocampus [94] − AD human temporal lobe neocortex [98] − ↑ 6-month-old APPswe/PSΔE9 mouse cerebral cortex [128] − AD human superior temporal neocortex [101] − ↑ 12-week AD rabbit frontal cortex [109]	− ↓ AD human cerebellum, hippocampus and medial frontal gyrus [96] − ↓ AD human cortex [97] − ↓ 3-month-old APPswe/PSΔE9 mouse cerebral cortex [128] − ↓ APP23 mouse hippocampus [99] − ↓ AD human temporal cortex [100] − ↓ 18-month-old rats [108] − ↓ 6-week AD rabbit frontal cortex [109]	− BACE1 [97] − TGFBI [104] − TRIM2 [104] − SIRT1 [104] − CAMKK2 [105]	− ROS [95] − Aβ [99]
**miR-124**	N/A	− ↓ AD human hippocampus (*p* > 0.05) [94] − ↓ AD human anterior temporal cortex [116] − ↓ AD human hippocampus [121]	− PTBP1 [116] − BACE1 [120]	− Aβ [120]
**miR-181**	N/A	− ↓ AD human cerebellum and hippocampus [96] − ↓ AD human cortex [97] − ↓ AD human parietal lobe cortex [124] − ↓ 7-month-old APP23 mouse hippocampus [99] − ↓ AD human temporal cortex [100]	− SPTLC1 [125] − TRIM2 [104] − SIRT1 (conflicting reports) [126] − BTBD3 [104] − HMG1 [126] − Bcl2 [126] − NAMPT [126] − MeCP2 [126] − XIAP [126]	− Aβ [99]
**miR-29**	− ↑ AD human medial frontal gyrus [96]	− ↓ AD human cortex [97] − ↓ 6-month-old APPswe/PSΔE9 mouse cerebral cortex [128] − ↓ AD human parietal lobe cortex [124] − ↓ AD human frontal cortex [130]	− BACE1 [97,129,139] − NAV3 [130] − Bim [134] − Bmf [134] − Hrk [134] − Puma [134] − N-Bak [134] − SPTLC2 [125]	N/A
**miR-34**	− ↑ AD human cerebellum, hippocampus and medial frontal gyrus [96] − ↑ 6-month-old APPswe/PSΔE9 mouse cerebral cortex [128] − ↑ 24-month-old C57B1/6J mouse hippocampus [147] − ↑ 24-month-old APPS1-21 mouse hippocampus [147]	N/A	− Bcl2 [128] − SIRT1 [147] − Tau [151]	N/A
**miR-107**	N/A	− ↓ AD human temporal cortex [154] − ↓ Tg19959 mouse brain [161] − ↓ AD human hippocampus [165]	− BACE1 [154,155] − GRN [158] − CF1 [161] − *CDK5R1* (encodes p35) [162] − ADAM10 [164]	− sAβ [166]
**miR-146**	− ↑ AD human hippocampus and neocortex [167] − Increase in AD human temporal lobe neocortex [98]	− ↓ AD human cerebellum, hippocampus and medial frontal gyrus [96]	N/A	− NF-κB [167] − IL-1β [167] − Aβ [99,167] − H_2_O_2_ [167] − TNFα [169] − ROS [171]
**miR-101**	N/A	− ↓ AD human anterior temporal cortex and cerebellum [97] − ↓ AD human parietal lobe cortex [124]	− APP [173,174]	N/A
**miR-195**	− ↑ AD human hippocampus [121]	− ↓ AD human CSF [96]	− BACE1 [175]	N/A
**miR-153**	N/A	− ↓ AD human frontal cortex [178]	− α-synuclein [176] − APP [178]	N/A
